# Perinatal mortality in the municipality of Panchimalco, San Salvador: a case series

**DOI:** 10.17843/rpmesp.2024.411.13335

**Published:** 2024-03-25

**Authors:** Evelyn Castellanos-Flores

**Affiliations:** 1 Ministerio de Salud, Instituto Nacional de Salud, San Salvador, El Salvador. Ministerio de Salud Instituto Nacional de Salud San Salvador El Salvador

**Keywords:** Perinatal Mortality, Fetal Death, Prenatal Care

## Abstract

Perinatal mortality is an indicator that reflects the impact of maternal and infant care in a country. This study presents nine cases of perinatal mortality that occurred in the municipality of Panchimalco, El Salvador. The information was obtained from audit reports. The mothers of the deceased infants were aged between 17 and 43 years, did not use contraceptive methods, had incomplete prenatal controls and averaged a gestational age of 31 weeks. Three deliveries were attended in the community. Most perinatal deaths occurred before delivery due to unknown causes, and live births were preterm. We identified factors such as deficits in comprehensive care for women. Further studies are needed to determine the main factors influencing perinatal deaths in El Salvador.

## INTRODUCTION

Perinatal mortality is one of the major health issues globally, it reflects the quality of healthcare provided to both the mother and the newborn ^1^. According to the World Health Organization (WHO) and the International Classification of Diseases (ICD-10), it encompasses fetal deaths from 22 weeks of pregnancy to deaths within the first week of life ^2^. In 2019, the mortality rate for this population worldwide was 4.3 million ^3^. Perinatal mortality is associated with various maternal-fetal risk factors and with social, cultural, and economic determinants ^4^.

According to the records of the Health Information System on Morbidity, Mortality, and Vital Statistics of the Ministry of Health in El Salvador (SIMMOW), during the period from 2018 to 2020, the average perinatal mortality rate was 11.5 per 1000 births ^5^. Considering that perinatal mortality is one of the most important issues related to comprehensive maternal and child care, there is limited scientific evidence on the subject and no publications describing the situation in El Salvador. This study aims to characterize sociodemographic and clinical aspects of maternal and child cases of perinatal deaths that occurred during the first year of the COVID-19 pandemic in a municipality south of San Salvador in El Salvador. The results obtained aim to contribute to the formulation of new strategies and research directions aimed at reducing this problem.

**KEY MESSAGES**

Motivation for the study: It is necessary to understand the maternal and infant characteristics of perinatal deaths. Additionally, it is required to generate evidence that contributes to a better understanding of these events.

Main findings: Nine cases of perinatal deaths with maternal-fetal risk characteristics were identified. Most deaths occurred before delivery, with prematurity predominating in the neonates.

Implications: Understanding the maternal and infant characteristics of perinatal deaths is essential for developing preventive strategies aimed at reducing risk factors related to perinatal mortality.

## THE STUDY

A case series study was conducted on all perinatal deaths that occurred in the municipality of Panchimalco during 2020. This municipality is located 17 km south of the capital, at an altitude of 600 meters above sea level, covering an area of 91 km^2^. According to estimates from 2020, its population was 41,260 habitants, with over 65% residing in rural areas. Panchimalco is recognized as an indigenous town, where the primary economic activity is the production of products derived from maize ^6^.

This study presents the characteristics of nine cases of deaths that occurred from the 22nd week of gestation to the first week of life, according to the WHO classification and the CIE-10 ^2^. In the first stage of the information-gathering process, we conducted a search in the SIMMOW ^5^ for cases diagnosed with perinatal death in the municipality of Panchimalco in 2020. In the second stage, these cases were cross-referenced with the audit reports of the perinatal death registration books from the two health units in the municipality. The audit reports contain clinical summaries, verbal autopsies, and interviews conducted with mothers by pediatricians, epidemiologists, and public health officials. The information gathered in the audit reports was used to define the diagnosis of perinatal death cases based on the CIE-10, taking into account codes P00 to P96, which refer to conditions originating in the perinatal period, and codes Q00-Q99 referring to congenital malformations, deformities, and chromosomal anomalies ^2^.

Data from SIMMOW and audit reports were collected using a digital form created with the KoBoToolbox tool ^7^. The variables considered were based on sociodemographic aspects, maternal clinical history, and characteristics of the deceased. The supplementary material provides a complete description of the study variables.

The resulting database was analyzed using Excel with the Real Statistics add-in. To assess the normality of numerical variables, the Shapiro-Wilk test was applied, revealing numerical variables with non-normal distribution. In these, the median and interquartile range (IQR) were used for their description. Additionally, the perinatal mortality rate was calculated as the number of perinatal deaths per 1000 births (both live and deceased), and tables were generated to present the main findings.

The research process was conducted confidentially and we adhered to the ethical principles of the Helsinki Declaration and good clinical practices ^8^. The project was approved by the Research Ethics Committee of the Metropolitan Health Region through the certificate 2021-CLEIS-09.

## FINDINGS

In the municipality of Panchimalco, in 2020, a total of nine cases of perinatal deaths were recorded out of 788 births (both live and deceased). This represents a perinatal mortality rate of 11.4 per 1000 births. The median age of the mothers was 28 years (IQR: 24.5-34.5).

Most (five cases) resided in rural areas, and the educational level (seven cases) was basic, with one mother having no education and another having completed high school. Additionally, no instances of maternal violence, drug use, or alcohol consumption were identified (Table 1).

**Table 1:** Sociodemographic characteristics of mothers who experienced perinatal deaths.

Cases	Education level	Family status	Occupation	Number of household members	Area of residence	Housing tenure	Basic services	Time to reach healthcare facility
Case 1	Elementary school	Accompanied	Homemaker	4	Rural	Owned	Sí	Less 1 h
Case 2	Bachelor’s degree	Accompanied	Homemaker	5	Urban	Rented	Sí	Less 1 h
Case 3	Elementary school	Accompanied	Homemaker	5	Urban	Borrowed	Sí	Less 1 h
Case 4	High school	Married	Homemaker	5	Rural	Owned	Sí	Less 1 h
Case 5	Elementary school	Accompanied	Homemaker	2	Rural	Owned	Sí	Less 1 h
Case 6	None	Accompanied	Homemaker	13	Urban	Borrowed	Sí	More 1 h
Case 7	High school	Accompanied	Informal employment	4	Rural	Rented	Sí	More 1 h
Case 8	Elementary school	Accompanied	Homemaker	3	Rural	Owned	Sí	More 1 h
Case 9	High school	Accompanied	Homemaker	6	Urban	Owned	Sí	Less 1 h

In the majority of cases (six), women had more than two pregnancies, being the most notable case the one numbered 6, who had nine pregnancies, the highest number recorded. Most mothers (six cases) did not report any history of previous illnesses; however, cases 1, 3, and 9 presented overweight and obesity, with case 3 additionally having vaginosis. No history of COVID-19 was described. Prenatal care attendance was irregular, with a median of 3 (IQR: 0.5-5.5) visits, all of which were performed at first-level facilities (Table 2).

**Table 2:** Key findings regarding cases of perinatal deaths.

Case	Maternal age (years)	Obstetric formula	History of illness	Number of prenatal check-ups	Birth care location	Condition at birth	Gestational age (weeks)	Birth weight (grams)	Sex of the deceased	Hours after birth	Cause of death diagnosis
1	27	G2P1001	Overweight	7	Hospital	Deceased	38	2645	Female	0	Unspecified fetal death
2	28	G1P0000	No	3	Hospital	Deceased	36	1770	Male	0	Unspecified fetal death
3	25	G4P3300	Overweight and vaginosis	6	Hospital	Deceased	35	2550	Male	0	Patau syndrome
4	24	G2P1001	No	0	Community	Deceased	26	1115	Female	0	Unspecified fetal death
5	36	G1P0000	No	3	Hospital	Deceased	27	709	Male	0	Unspecified fetal death
6	43	G9P8008	No	0	Hospital	Alive	33	900	Male	3	Edwards syndrome
7	17	G1P0000	No	1	Community	Alive	27	1001	Male	3	Extreme prematurity
8	29	G2P1001	No	4	Community	Alive	22	600	Female	8	Neonatal bacterial sepsis
9	33	G2P1001	Obesity	5	Hospital	Alive	36	2635	Male	2	Birth asphyxia

According to the delivery care, we observed that the hospital was the main place of delivery (six cases), with three cases delivered in the community. The gestational age had a median of 33 (IQR: 26.5-36.0) weeks. Regarding birth weight, six cases were under 2500 g (Table 2).

The diagnoses were stillbirths (four cases), and were classified as unspecified fetal deaths. One case was registered as death due to congenital malformation. In early neonatal deaths, one case was classified as due to congenital malformation, surviving three hours; another due to asphyxia, surviving two hours; one due to unspecified bacterial sepsis, surviving eight hours; and another due to extreme prematurity (2nd twin), surviving three hours (Table 2).

In other findings, the majority of parents (eight cases) did not use contraceptive methods. Regarding previous perinatal death history, only case 3 were registered as such.

The duration of labor was less than 12 hours in seven cases, and in two cases (3 and 4), it exceeded 12 hours. The majority of cases (seven) had membrane rupture at the time of delivery. Six cases were delivered vaginally, and three cases were delivered by cesarean section, with cases 1 and 3 due to previous cesarean sections and case 9 due to transverse position cesarean. Male sex predominated in the majority of cases (six cases) (Table 3).

**Table 3:** Maternal-fetal characteristics of perinatal death cases.

Cases	History of perinatal death	Previous contraception	Planned pregnancy	Location of prenatal care	Transferred for childbirth care	Time until birth	Moment of membrane rupture	Birth route	Birth results
Case 1	No	No	Yes	Primary care level	Yes	Less 12 h	During childbirth	Cesarea	Only one
Case 2	No	No data	Yes	Primary care level	No	Less 12 h	During childbirth	Vaginal	Only one
Case 3	Yes	No	Yes	Primary care level	Yes	More 12 h	During childbirth	Cesarea	Only one
Case 4	No	No	Yes	None	No	More 12 h	Unknown	Vaginal	Only one
Case 5	No	No	No	Primary care level	No	Less 12 h	Unknown	Vaginal	Only one
Case 6	No	No	Yes	None	No	Less 12 h	During childbirth	Vaginal	Only one
Case 7	No	No	No	Primary care level	Yes	Less 12 h	During childbirth	Vaginal	Twin
Case 8	No	No	No	Primary care level	Yes	Less 12 h	During childbirth	Vaginal	Only one
Case 9	No	Yes	Yes	Primary care level	Yes	Less 12 h	During childbirth	Cesarea	Only one

In the reports, we found that the majority of delays (eight cases) corresponded to delay 1 (delay in seeking medical attention), while 3 cases (4, 7, and 8) corresponded to delay 2 (delay in reaching the nearest health facility), with no report of delay in case 3.

## DISCUSSION

The study reveals that the perinatal mortality rate in the municipality of Panchimalco in 2020 was 11.4 per 1000 births, exceeding the national rate (8.8 per 1000 births) ^5^. This municipality is predominantly rural and characterized by extreme poverty, sharing similarities with other studies conducted in high-risk areas for perinatal mortality ^9^.

In the nine reported cases, notable factors include rural origin, basic educational level, extreme maternal ages, multigravidity, diseases such as overweight, obesity, and vaginosis, non-compliance with prenatal care, delivery in the community, low birth weight for gestational age, and prematurity.

Some studies link the absence or low level of education with poor social development, leading to inadequate knowledge about maternal nutrition and health ^10^. Biological, social, and fetal pathology risk factors are related to extreme ages ^11^. We found a mother under 20 years old and two mothers over 35 years old. Another characteristic considered an obstetric risk factor in this study is multigravidity, found in the majority of cases ^12^.

In this study, three mothers had maternal conditions related to overweight, obesity, and vaginosis, with these cases corresponding to cesarean deliveries and birth weights over 2500 g. Systematic reviews indicate that these characteristics contribute to early placental and fetal dysfunction ^13^. Although national protocols for prenatal care recommend prenatal enrollment and six subsequent check-ups ^14^, this study reflects non-compliance with the number and regularity of check-ups, which according to some studies, contributes to the late identification of maternal and fetal pathologies ^15^.

Perinatal mortality is higher in out-of-hospital deliveries due to lack of adequate care ^16^, as evidenced by three deliveries attended in the community. A predominance of male sex was observed, considered more vulnerable to intrauterine stressors ^9^.

Low gestational age, low birth weight for gestational age, and prematurity are related to obstetric and healthcare factors ^17^, being common findings in this study, both in stillbirths as well as in neonates. Additionally, we found that all early neonatal deaths were prematures with short survival, a characteristic considered risk factors for perinatal death ^18^.

Regarding the increase in COVID-19 cases in El Salvador in 2020, none of the mothers in the study presented symptoms, and they reported no contact with sick individuals. According to a study conducted on mothers with COVID-19, the majority were asymptomatic ^19^. Additionally, the mental health of the mothers related to the stress of isolation and fear of contagion from leaving home was not assessed, a factor that is described as potentially related to prematurity ^20^.

In the study limitations, we must acknowledge that, due to the use of reports from healthcare facilities, there was no direct control over data collection from the mothers. Although the audit analysis identified delays, the attitudes, limitations in seeking immediate healthcare, transportation difficulties, as well as information regarding hospital management, could not be specified. Another limitation is the lack of RT-PCR tests for mothers to rule out SARS-CoV-2 infection, and in cases of unknown causes of death, no pathological examination (autopsy) was performed. On the other hand, this study helps to understand the behavior of perinatal deaths in a municipality of the country and describes the factors that may be related to these deaths.

In conclusion, perinatal mortality cases exhibit multifactorial characteristics in rural areas with limited socioeconomic resources, most of which correspond to deaths occurring before delivery of unknown cause, and those born alive commonly had prematurity as a factor for subsequent death. The majority of mothers had high-risk pregnancies, and the diagnoses of deaths are related to preventable causes, possibly linked to delays.

There is a need to strengthen comprehensive care for women from early ages, during the prenatal, delivery, postpartum, and immediate newborn care periods. Given that this is a study conducted in one municipality of the country, we consider it necessary to develop further research that allows for a deeper understanding of the factors related to perinatal deaths. It is necessary to incorporate autopsies into perinatal mortality surveillance protocols, especially in cases of unknown origin.
