# Supplementary material for: Perinatal mortality in the municipality of Panchimalco, San Salvador: a case series
Source: Rev Peru Med Exp Salud Publica. 2024 Mar 25;41(1):83–8. doi: 10.17843/rpmesp.2024.411.13335 (PMC11149758; doi:10.17843/rpmesp.2024.411.13335)
Supplement: Supplementary material. — Available in the electronic version of the RPMESP. [file rpmesp-41-01-13335-s001.pdf]

MATERIAL SUPLEMENTARIO

Tabla suplementaria 1. Definición de variables de estudio.

Categoría	Variable	Definición	Dimensión
Sociodemográficos	Edad	Años cumplidos por la madre	17 - 49
	Educación	Último grado académico alcanzado por la madre	Primara, secundaria, bachillerato
	Estado familiar	Tipo de unión conyugal	Soltera, casada
	Ocupación	Tipo de empleo que realiza actualmente	Formal, informal, ama de casa
	Miembros del grupo familiar	Número de miembros de la familia con quienes convive	1-10
	Área	Grupo de población del que procede	Urbano, rural
	Servicios básicos	Servicios esenciales necesarios para el desarrollo, seguridad y bienestar	Agua, luz, teléfono
	Accesibilidad a los servicios de salud	Tiempo que tarda en llegar al establecimiento de salud más cercano desde su hogar	<1 hora, > 1 hora
Antecedentes clínicos de las embarazadas	Formula obstétrica	Fórmula que expresa el número de embarazos, partos, prematuros, abortos y vivos	GPPAV
	Periodo intergenésico	Tiempo transcurrido desde el último embarazo y el inicio del siguiente	< 2 años, > 2 años
	Antecedentes de muerte perinatal	Número de muertes fetales a partir de las 22 semanas y neonatales tempranas previas	Si, no
	Anticoncepción	Utilización de métodos de planificación familiar	Si, no
	Controles prenatales	Número de atenciones preventivas de la embarazada	0-7
	Nivel atención prenatal	Nivel de atención de los controles prenatales	Ninguno, primer nivel**, segundo nivel y tercer nivel***
	Antecedentes de enfermedades de las embarazadas	Nombre de la enfermedad de la madre durante el embarazo	Sobrepeso, obesidad, vaginosis, ninguna
	Referencia	Indicación en el centro de salud para recibir atención de parto en un hospital	Si, no
	Duración del trabajo de parto	Tiempo que dura o transcurre entre el comienzo y el fin de un proceso del parto	< 12 horas, > 12 horas
	Momento de ruptura de membranas	Tiempo que transcurrió entre la ruptura del saco amniótico y el parto	No sabe, antes del parto, durante el parto
	Lugar de atención del parto	Centro de salud donde se atendió el parto	Hospital, comunitario
	Vía del parto	Vía del parto a través de la vagina o abdomen	Vaginal, cesárea
	Número de productos del parto	Número de fetos vivos o muertos al momento del parto	Único, múltiple
Características de los fallecidos	Estado al nacer	Condición del feto	Vivo, muerto
	Edad gestacional	Semanas de embarazo al momento del parto	22-40
	Sexo/feto o recién nacido	Características biológicas y fisiológicas que definen al hombre y la mujer	Masculino, femenino
	Peso al nacer	Peso en gramos al nacimiento	500 - 2500
	Horas de vida	Tiempo de vida desde nacimiento hasta defunción	0 - 12
	Diagnóstico de muerte	Clasificación del diagnóstico según CIE-10*	Muerte fetal de causa no especificada, Sospecha de síndrome de Patau, sospecha de síndrome Edwards, Sepsis bacteriana de recién nacido, Asfixia del nacimiento, Inmaturidad extrema
Demoras en la atención	Demora 1	Tardanza en buscar atención medica	Si, no
	Demora 2	Tardanza para trasladarse al establecimiento de salud más cercano	Si, no
	Demora 3	Atención brindada	Si, no

*CIE-10 = Clasificación internacional de enfermedades / ** Unidades de salud, clínicas comunales, casas de salud; *** Hospitales generales y especializados

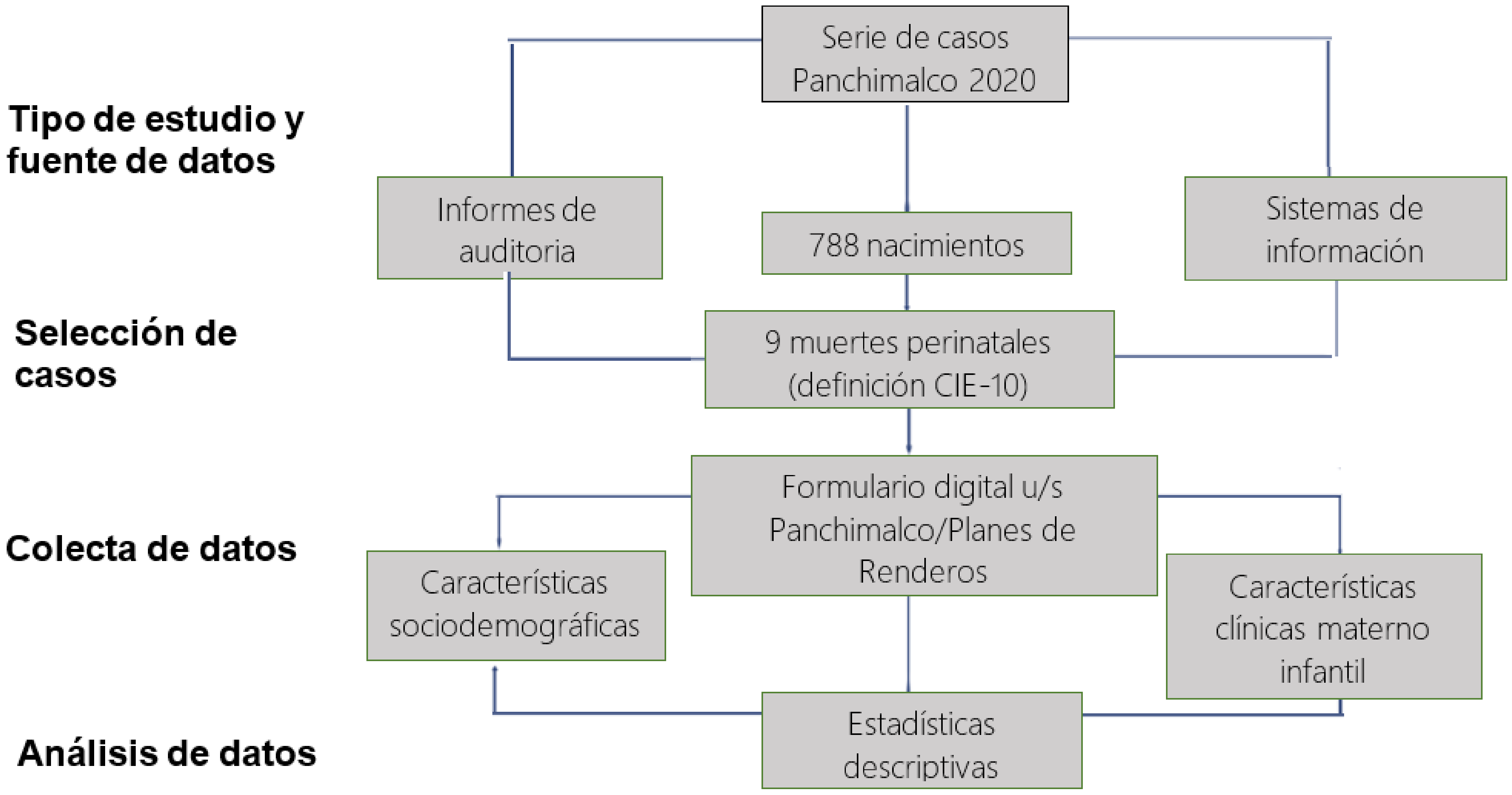

Figura suplementaria 1. Metodología.
