# Supplementary material for: Perinatal mortality in the municipality of Panchimalco, San Salvador: a case series
Source: Rev Peru Med Exp Salud Publica. 2024 Mar 25;41(1):83–8. doi: 10.17843/rpmesp.2024.411.13335 (PMC11149758; doi:10.17843/rpmesp.2024.411.13335)
Supplement: Translation

# Translation: Mortalidad perinatal en el municipio de Panchimalco, San Salvador: una serie de casos

**Authors:** Evelyn Castellanos-Flores

**Affiliations:** 1 Ministerio de Salud, Instituto Nacional de Salud, San Salvador, El Salvador.

**Keywords:** Mortalidad Perinatal, Muerte Fetal, Atención Prenatal

## Abstract

La mortalidad perinatal es un indicador que refleja el impacto de la atención materno-infantil de un país. Este estudio presenta nueve casos de la mortalidad perinatal ocurridos en el municipio de Panchimalco, El Salvador. La información se obtuvo de los informes de auditorías. Las madres de los fallecidos tenían edades entre 17 a 43 años, sin uso de anticonceptivos, con controles prenatales incompletos y un promedio de edad gestacional de 31 semanas, tres partos fueron atendidos en la comunidad. La mayoría de muertes perinatales ocurrieron antes del parto de causa desconocida y los nacidos vivos fueron prematuros. Se identificaron factores como el déficit en la atención integral a la mujer. Se requiere de nuevos estudios para determinar los principales factores que influyen en las muertes perinatales en El Salvador.

## INTRODUCCIÓN

La mortalidad perinatal es uno de los principales problemas de salud a nivel mundial, refleja la calidad de atención en salud que se brinda a la madre y al recién nacido ^1^. Según la Organización Mundial de la Salud (OMS) y la clasificación internacional de enfermedades (CIE-10), comprende las muertes fetales desde las 22 semanas de gestación hasta las muertes en la primera semana de vida ^2^. Para el 2019 la mortalidad de esta población en el mundo fue de 4,3 millones ^3^. La mortalidad perinatal está relacionada con diferentes factores de riesgo materno-fetales y con determinantes sociales, culturales y económicos ^4^.

Según los registros del Sistema de Información en Salud, sobre Morbimortalidad y Estadísticas Vitales del Ministerio de Salud en El Salvador (SIMMOW), durante el periodo 2018 al 2020, la tasa promedio de mortalidad perinatal fue de 11,5 por 1000 nacimientos ^5^. Considerando que la mortalidad perinatal es uno de los problemas más importantes relacionados a la atención integral materno-infantil, hay poca evidencia científica sobre el tema y no existen publicaciones que describan la situación en El Salvador. El objetivo de este estudio es caracterizar aspectos sociodemográficos y clínicos materno-infantiles de una serie de casos de muertes perinatales ocurridos en el primer año de la pandemia de la COVID-19, en un municipio al sur de San Salvador en El Salvador. Con los resultados obtenidos se pretende contribuir a la formulación de nuevas estrategias y líneas de investigación dirigidas a disminuir este problema.

**MENSAJE CLAVE**

Motivación para realizar el estudio: Es necesario conocer las características maternas e infantiles de las muertes perinatales. Además, es fundamental generar evidencia que contribuya a una mejor comprensión de estos eventos.

Principales hallazgos: Se identificaron nueve casos de muertes perinatales y con características materno-fetales de riesgo. La mayoría de las muertes ocurrieron antes del parto y predominó la prematurez en los neonatos.

Implicancias: El comprender las características materno-infantiles de las muertes perinatales, es esencial para desarrollar estrategias preventivas orientadas a disminuir los factores de riesgo relacionados con la mortalidad perinatal.

## EL ESTUDIO

Se realizó un estudio de serie de casos del total de muertes perinatales ocurridas en el municipio de Panchimalco, durante el 2020. El municipio de Panchimalco está situado a 17 km al sur de la capital, a una altitud de 600 m s. n. m, con una extensión territorial de 91 km^2^. Según estimaciones del 2020, su población era de 41 260 habitantes, con más del 65% residiendo en áreas rurales. Panchimalco es reconocido como un pueblo indígena, donde la principal actividad económica es la producción de derivados del maíz ^6^.

Este estudio muestra las características de nueve casos de defunciones que ocurrieron desde la semana 22 de gestación hasta la primera semana de vida, según la clasificación de la OMS y el CIE-10 ^2^. En la primera etapa del proceso de obtención de la información, se efectuó una búsqueda en el SIMMOW ^5^ de los casos con diagnóstico de muerte perinatal del municipio de Panchimalco en el 2020. En la segunda etapa se corroboraron estos casos con los informes de auditoría de los libros de registro de muertes perinatales de las dos unidades de salud del municipio. Los informes de auditoría contienen el resumen clínico, la autopsia verbal y la entrevista realizada a las madres por parte de los pediatras, epidemiólogos y salubristas. La información recabada en los informes de auditoría es utilizada para definir el diagnóstico de los casos de muertes perinatales basado en la CIE-10, tomando en cuenta los códigos de P00 al P96, que hace referencia a las afecciones originadas en el periodo perinatal y los códigos de Q00-Q99 referente a las malformaciones congénitas, deformidades y anomalías cromosómicas ^2^.

Mediante un formulario digital creado con la herramienta KoBoToolbox ^7^ se recabaron los datos del SIMMOW e informes de auditorías. Las variables que se tomaron en cuenta se basaron en aspectos sociodemográficos, antecedentes clínicos de las madres y características de los fallecidos. En el material suplementario se muestra la descripción completa de las variables de estudio.

La base de datos resultante fue analizada en forma descriptiva con el programa Excel con complemento Real Statistics. Para verificar la normalidad de las variables numéricas, se aplicó la prueba de Shapiro-Wilk, encontrando variables numéricas sin distribución normal, por lo que se utilizó la mediana y el rango intercuartílico (RIC) para su descripción. Además, se calculó la tasa de mortalidad perinatal como el número de muertes perinatales por cada 1000 nacimientos (tanto vivos como fallecidos) y se elaboraron tablas con los principales hallazgos.

El proceso de investigación se desarrolló bajo confidencialidad y se realizó cumpliendo los principios éticos de la declaración de Helsinki y buenas prácticas clínicas ^8^. El proyecto fue aprobado por el Comité de Ética de Investigación de la Región Metropolitana de Salud a través de la constancia 2021-CLEIS-09.

## HALLAZGOS

En el municipio de Panchimalco, durante el 2020, se registraron un total de nueve casos de muertes perinatales, de 788 nacimientos (tanto vivos como fallecidos). Esto representa una tasa de mortalidad perinatal de 11,4 por cada 1000 nacimientos. La mediana de la edad de las madres fue de 28 años (RIC: 24,5-34,5).

El área de residencia en la mayoría (cinco casos) corresponde al área rural y el nivel educativo (siete casos) fue básico, en una madre ninguno y en otra hasta bachillerato. Además, no se identificó que las madres sufrieran violencia, ni que ingirieran drogas o alcohol (Tabla 1).

**Tabla 1:** Características sociodemográficas de las madres que presentaron muertes perinatales.

Casos	Escolaridad	Estado familiar	Ocupación	Núm. de miembros del hogar	Área de residencia	Tenencia de vivienda	Servicios básicos	Tiempo para llegar a establecimiento de salud
Caso 1	Primaria	Acompañada	Ama de casa	4	Rural	Propia	Sí	Menor 1 h
Caso 2	Bachillerato	Acompañada	Ama de casa	5	Urbana	Alquilada	Sí	Menor 1 h
Caso 3	Primaria	Acompañada	Ama de casa	5	Urbana	Prestada	Sí	Menor 1 h
Caso 4	Secundaria	Casada	Ama de casa	5	Rural	Propia	Sí	Menor 1 h
Caso 5	Primaria	Acompañada	Ama de casa	2	Rural	Propia	Sí	Menor 1 h
Caso 6	Ninguna	Acompañada	Ama de casa	13	Urbana	Prestada	Sí	Mayor de 1 h
Caso 7	Secundaria	Acompañada	Empleo informal	4	Rural	Alquilada	Sí	Mayor de 1 h
Caso 8	Primaria	Acompañada	Ama de casa	3	Rural	Propia	Sí	Mayor de 1 h
Caso 9	Secundaria	Acompañada	Ama de casa	6	Urbana	Propia	Sí	Menor 1 h

En la mayoría de los casos (seis), se observa que las mujeres tuvieron más de dos embarazos. Siendo más notable en el caso 6, que presentó nueve embarazos, correspondiendo al mayor número de embarazos registrados. La mayoría de las madres (seis casos), no reportaron antecedentes de enfermedades, sin embargo, los casos 1, 3 y 9 presentaron sobrepeso y obesidad; el caso 3 tiene agregada una vaginosis. No se describen antecedentes de COVID-19. La asistencia a controles prenatales fue de forma irregular, con una mediana de 3 (RIC: 0,5-5,5) controles, los cuales fueron atendidos en establecimientos del primer nivel (Tabla 2).

**Tabla 2:** Principales hallazgos sobre los casos de muertes perinatales.

Caso	Edad materna (años)	Fórmula obstétrica	Antecedente de enfermedad	Núm. de controles prenatales	Lugar de atención del parto	Estado al nacer	Edad gestacional (semanas)	Peso al nacer (gramos)	Sexo del fallecido	Horas de nacido	Diagnóstico de muerte
1	27	G2P1001	Sobrepeso	7	Hospital	Muerto	38	2645	Femenino	0	Muerte fetal de causa no especificada
2	28	G1P0000	No	3	Hospital	Muerto	36	1770	Masculino	0	Muerte fetal de causa no especificada
3	25	G4P3300	Sobrepeso y vaginosis	6	Hospital	Muerto	35	2550	Masculino	0	Síndrome de Patau
4	24	G2P1001	No	0	Comunidad	Muerto	26	1115	Femenino	0	Muerte fetal de causa no especificada
5	36	G1P0000	No	3	Hospital	Muerto	27	709	Masculino	0	Muerte fetal de causa no especificada
6	43	G9P8008	No	0	Hospital	Vivo	33	900	Masculino	3	Síndrome de Edwards
7	17	G1P0000	No	1	Comunidad	Vivo	27	1001	Masculino	3	Inmaturidad extrema
8	29	G2P1001	No	4	Comunidad	Vivo	22	600	Femenino	8	Sepsis bacteriana del recién nacido
9	33	G2P1001	Obesidad	5	Hospital	Vivo	36	2635	Masculino	2	Asfixia del nacimiento

De acuerdo con la atención de los partos, se observó que el hospital fue el principal lugar de atención (seis casos) y que tres casos fueron atendidos en la comunidad. La edad gestacional tuvo una mediana de 33 (RIC: 26,5-36,0) semanas. En relación con el peso al nacer, seis casos fueron menores de 2500 g (Tabla 2).

Los diagnósticos en los mortinatos (cuatro casos) se clasificaron como muertes fetales no especificadas y un caso como muerte por malformación congénita, en las muertes neonatales tempranas se clasificó un caso por malformación congénita, que vivió tres horas, otro por asfixia, que vivió dos horas, otro por sepsis bacteriana no especificada, que vivió ocho horas y otro por inmaturidad extrema (2^o^ gemelo) que vivió tres horas (Tabla 2).

En otros hallazgos, la mayoría de los padres (ocho casos) no utilizaron métodos anticonceptivos. En relación con los antecedentes de muerte perinatal, solo el caso 3 presentó un historial previo de este tipo de característica.

La duración del trabajo de parto en siete casos fue menor de 12 horas y en dos casos (3 y 4) fue mayor de 12 horas. La mayoría de los casos (siete) presentaron ruptura de membranas en el momento del parto. En seis casos los partos fueron por vía vaginal y en tres casos por cesárea, el caso 1 y 3 por cesárea anterior y el caso 9 por cesárea por posición transversa. Predominando en la mayoría (seis casos) el sexo masculino (Tabla 3).

**Tabla 3:** Características materno-fetales de los casos de muerte perinatales.

Casos	Antecedentes de muerte perinatal	Anticoncepción previa	Embarazo deseado	Lugar de control prenatal	Referencia para atención del parto	Duración del trabajo de parto	Momento de ruptura de membranas	Vía del parto	Productos del parto
Caso 1	No	No	Sí	Primer nivel de atención	Sí	Menor 12 h	Durante el parto	Cesárea	Único
Caso 2	No	No dato	Sí	Primer nivel de atención	No	Menor 12 h	Durante el parto	Vaginal	Único
Caso 3	Sí	No	Sí	Primer nivel de atención	Sí	Mayor 12 h	Durante el parto	Cesárea	Único
Caso 4	No	No	Sí	Ninguno	No	Mayor 12 h	No sabe	Vaginal	Único
Caso 5	No	No	No	Primer nivel de atención	No	Menor 12 h	No sabe	Vaginal	Único
Caso 6	No	No	Sí	Ninguno	No	Menor 12 h	Durante el parto	Vaginal	Único
Caso 7	No	No	No	Primer nivel de atención	Sí	Menor 12 h	Durante el parto	Vaginal	Gemelar
Caso 8	No	No	No	Primer nivel de atención	Sí	Menor 12 h	Durante el parto	Vaginal	Único
Caso 9	No	Sí	Sí	Primer nivel de atención	Sí	Menor 12 h	Durante el parto	Cesárea	Único

Se encontró en los informes que la mayoría de las demoras (ocho casos), corresponden a la demora 1 (tardanza en buscar atención medica), 3 casos (4, 7 y 8) corresponden a la demora 2 (tardanza para trasladarse al establecimiento de salud más cercano) y no hay reporte de demora en el caso 3.

## DISCUSIÓN

El estudio muestra que la tasa de mortalidad perinatal del municipio de Panchimalco para el año 2020 fue de 11,4 por cada 1000 nacimientos, que supera la tasa nacional (8,8 por cada 1000 nacimientos) ^5^. Este municipio se caracteriza por ser predominantemente rural y de extrema pobreza, compartiendo similitudes con otros estudios de lugares de riesgo de mortalidad perinatal ^9^.

En los nueve casos reportados destacan la procedencia rural, el nivel educativo básico, las edades maternas extremas, madres multigestas, enfermedades con sobrepeso, obesidad y vaginosis, el incumplimiento de controles prenatales, la atención de partos en la comunidad, bajo peso para edad gestacional y la prematuridad.

Algunos estudios relacionan el nulo o bajo nivel educativo encontrado con un pobre desarrollo social, que conlleva un deficiente conocimiento sobre la nutrición y salud materna ^10^. Los factores de riesgo biológico, social y de patología fetal, están relacionados a edades extremas ^11^. Entre los hallazgos de este estudio se encontró una madre menor de 20 años y dos madres mayores de 35 años. Otra característica considerada de riesgo obstétrico en este estudio son las madres multigestas, encontradas en la mayoría de los casos ^(^^12^.

En este estudio, tres madres presentaron enfermedades maternas, relacionadas con sobrepeso, obesidad y vaginosis, correspondiendo estos casos a partos por cesáreas y peso de los nacidos mayor de 2500 g. Revisiones sistemáticas indican que estas características contribuyen a la disfunción placentaria y fetal temprana ^13^. Aunque los protocolos nacionales de atención en el periodo prenatal, sugieren inscripción prenatal y seis controles subsecuentes ^14^. Este estudio refleja un incumplimiento en el número y regularidad de los controles, lo que según algunos estudios contribuye a la identificación tardía de patologías maternas y fetales ^15^.

La mortalidad perinatal es más alta en partos fuera del hospital, debido a la falta de una atención adecuada ^16^, como se evidencio en tres partos atendidos en la comunidad. Se observó un predominio del sexo masculino, considerado más vulnerable a factores estresantes en útero ^9^.

La baja edad gestacional, bajo peso para la edad gestacional y la prematurez se relacionan con factores obstétricos y de cuidados de la salud ^17^, siendo hallazgos comunes en este estudio tanto en mortinatos como en neonatos. Además, se encontró que el total de muertes neonatales tempranas fueron prematuros con supervivencia corta, características consideradas de riesgo de muerte perinatal ^18^.

En relación con el aumento de casos de COVID-19 en El Salvador en el 2020, ninguna madre del estudio presento síntomas, desconociendo contacto con personas enfermas. Según un estudio realizado en madres con COVID-19, encontraron que la mayoría eran asintomáticas ^19^. Además, no se evaluó la salud mental de la madre relacionada con el estrés del aislamiento y el temor al contagio por salir de casa, factor que se describe que puede estar relacionado con la prematurez ^20^.

En las limitaciones del estudio se debe reconocer que, debido al uso de informes de los establecimientos de salud, no se tuvo control directo de la recolección de estos datos a las madres. Aunque el análisis de auditoría detecta demoras, no se pudo conocer las actitudes y limitaciones para buscar la atención de salud de forma inmediata y de todas las dificultades para trasladarse, así como la información referente al manejo hospitalario. Otra limitación es que no se llevaron a cabo pruebas de RT-PCR a las madres, para descartar infección por SARS-CoV-2, y en aquellas muertes de causas desconocidas, no se realizó un examen patológico (autopsia). Por otro lado, este estudio ayuda a conocer el comportamiento de las muertes perinatales en un municipio del país y describe los factores que podrían estar relacionados a las muertes.

En conclusión, los casos de mortalidad perinatal presentan características multifactoriales en áreas rurales de escasos recursos socioeconómicos, las cuales corresponden en su mayoría a muertes ocurridas antes del parto de causa desconocida, y los que nacieron vivos, tuvieron como factor común la prematurez. Se halló que la mayoría de las madres tenían embarazos de riesgo y que los diagnósticos de muertes se relacionan con causas prevenibles, posiblemente vinculadas a demoras.

Es necesario el fortalecimiento de la atención integral a la mujer desde edades tempranas, así como en el periodo prenatal, parto, posparto y la atención inmediata del recién nacido. Por ser un estudio en un municipio del país, consideramos necesario el desarrollo de nuevos trabajos que permitan ampliar y comprender a mayor profundidad los factores relacionados a las muertes perinatales. Es esencial incorporar la autopsia en los protocolos de vigilancia de mortalidad perinatal, sobre todo en las muertes de origen desconocido.
